# Trace elements determination in seawater by ICP-MS with on-line pre-concentration on a Chelex-100 column using a ‘standard’ instrument setup.

**DOI:** 10.1016/j.mex.2015.06.003

**Published:** 2015-06-18

**Authors:** Jens Søndergaard, Gert Asmund, Martin M. Larsen

**Affiliations:** Department of Bioscience, Aarhus University, Frederiksborgvej 399, DK-4000 Roskilde, Denmark

**Keywords:** Trace elements determination in seawater by ICP-MS with on-line pre-concentration on a Chelex-100 column using a ‘standard’ instrument setup, Trace elements, Seawater analysis, ICP-MS, Chelex-100, On-line pre-concentration

## Abstract

Trace element determination in seawater is analytically challenging due to the typically very low concentrations of the trace elements and the potential interference of the salt matrix. A common way to address the challenge is to pre-concentrate the trace elements on a chelating resin, then rinse the matrix elements from the resin and subsequently elute and detect the trace elements using inductively coupled plasma mass spectrometry (ICP-MS). This technique typically involves time-consuming pre-treatment of the samples for ‘off-line’ analyses or complicated sample introduction systems involving several pumps and valves for ‘on-line’ analyses. As an alternative, the following method offers a simple method for ‘on-line’ analyses of seawater by ICP-MS. As opposed to previous methods, excess seawater was pumped through the nebulizer of the ICP-MS during the pre-concentration step but the gas flow was adjusted so that the seawater was pumped out as waste without being sprayed into the instrument. Advantages of the method include:

• Simple and convenient analyses of seawater requiring no changes to the ‘standard’ sample introduction system except from a resin-filled micro-column connected to the sample tube. The ‘standard’ sample introduction system refers to that used for routine digest-solution analyses of biota and sediment by ICP-MS using only one peristaltic pump; and
• Accurate determination of the elements V, Mn, Co, Ni, Cu, Zn, Cd and Pb in a range of different seawater matrices verified by participation in 6 successive rounds of the international laboratory intercalibration program QUASIMEME.

## Graphical abstract

**Figure:**
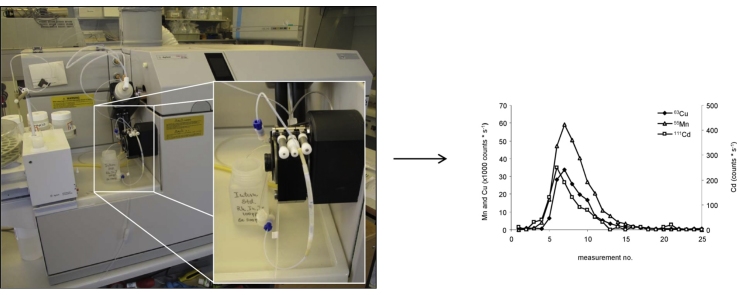

## Method details

### Instrumentation

All measurements were made using an Agilent 7500ce quadrupole-type ICP-MS equipped with a Babington nebulizer. The Babington nebulizer is especially suited for complex matrix samples and samples containing high concentrations of dissolved salts such as seawater. Standard nickel skimmer and sample cones were used.

Samples and standards were pumped through a 50 mm long and 3 mm inner diameter (i.d.) chromatographic borosilicate glass column from Omnifit containing Chelex-100 resin. The preparation of the column is described in detail below. The column was placed between the peristaltic pump of the instrument and a 3-port Teflon connector piece where sample water was mixed on-line with internal standard (IS) solution before entering the nebulizer. ^72^Ge, ^103^Rh and ^193^Ir were used as IS to correct for variations in pump speed, plasma temperature, mass-specific instrument sensitivity etc. Standard Tygon PVC tubing (Agilent) was used for sampling tubes (1.02 mm i.d.) and IS tubes (0.19 mm i.d.).

Two separate instrument tune files were made for the pre-concentration and elution modes, respectively and the instrumental operating parameters are listed in Table 1. Torch box positioning, ion lens voltages, detector settings, sample depth and He gas flow were optimized daily using a 10 μg L^−1^ tuning solution of Li, Y, Ce and Tl. The instrument was tuned for robustness rather than maximum sensitivity to increase the resistance to matrix suppression effects (oxide levels were adjusted to <1%). In pre-concentration mode the nebulizer carrier gas flow was set to zero and the nebulizer makeup gas flow was adjusted so low (0.3 L Ar min^−1^) that only negligible amounts of the sample were sprayed into the instrument but high enough to consistently rinse the nebulizer. Excess water was led to waste automatically. In elution mode the nebulizer gas flows were set to normal operating levels and He was used as a collision gas to minimize interferences from polyatomic species (one of the key features of the Agilent 7500).

**Fig. 1:**
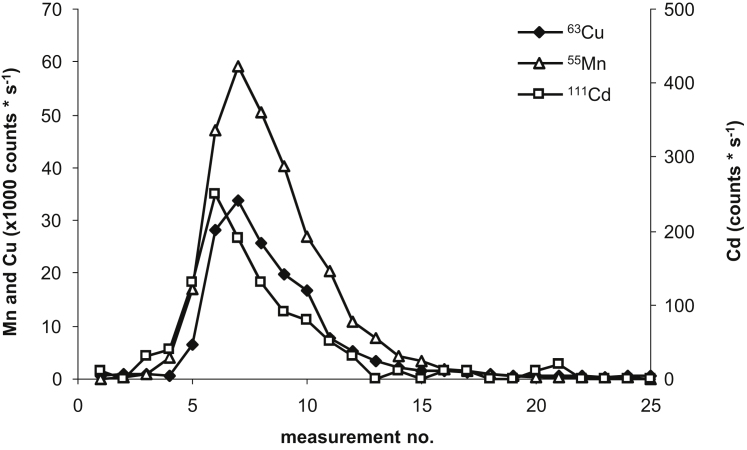
Elution profiles for the selected trace elements Mn, Cu and Cd in a sample of the certified seawater reference material SLEW-3 (Mn:1.61 μg L^−1^; Cu:1.55 μg L^−1^; Cd: 0.048 μg L^−1^). There are ∼7 s between each measuring point.

Applying the two tune files, two different method-programs were made for the pre-concentration and elution modes, respectively, and the settings are summarized in Table 2. Using Agilent’s ChemStation software the pre-concentration and elution modes were run as two consecutive samples applying the respective methods-programs. In pre-concentration mode the seawater sample was pumped through the column and only ^193^Ir was measured to check that only negligible amount of sample was sprayed into the instrument. After that a 0.05 M ammonium acetate buffer solution (pH 7.0) was run through the column in order to rinse it and flush it from loosely bound matrix products, primarily Na [1]. In the following elution mode 5% nitric acid was used as elutant and all the selected isotopes of the analyte elements and internal standards were measured (Table 1). More acid solution was then run through the column to rinse it thoroughly and finally the column was reconditioned using the 0.05 M ammonium acetate solution. The exact time-settings in the elution program are critical and depend on the number of masses analyzed as well as the specific instrument setup. Each time the setup is changed the program has to be adjusted so that the elution peak comes out after a few ‘baseline’ measurements (Fig. 1). The total measuring time using the proposed method was 655 s or 11 min.

### Reagents, standards and sample preparation

The laboratory-made Chelex-100 columns were prepared by making a slurry of 1 g Chelex-100 resin (Na-form; 100–200 mesh) in 1 M NH_4_OH and loading it into the column using a syringe until perfectly packed. Prior to this, the resin was cleaned by soaking it in 5 M HCl overnight before it was collected on a glass microfiber filter and further cleaned with 2 M HNO_3_ and Milli-Q water. Glass wool was placed in the ends of the column. Before the column was used, it was conditioned with 1 M ammonium acetate buffer solution (pH 7.0) in order to convert the active sites of the resin into ammonium form and raise the pH in the column to above pH 5 [2].

A 2 M stock solution of ammonium acetate was then prepared by diluting ∼140 g of 25–35% (m/m) analytical grade ammonia hydroxide (Merck) and ∼121 g of 100% Suprapure acetic acid (Merck) in 1000 mL of Milli-Q water. The pH was adjusted to 7.0 by adding either ammonia hydroxide or acetic acid. Last, the ammonium acetate buffer solution was purified by passing it through a 100 mm Chelex-100 filled glass column. From the purified stock buffer solution a 0.05 M ammonium acetate buffer solution was prepared by diluting the stock solution 1:40 with Milli-Q water. All Milli-Q water (18 MΩ cm^−1^) was purified in a Milli-Q water purification system (Millipore, Bedford, MA).

For the quantitative analysis, several standard solutions were prepared using the certified seawater reference material (CRM) NASS-5 with known quantities of Agilent multi-element calibration standard-2A added giving final concentrations of 0, 0.2, 0.5, 1, 2, 5 and 10 μg L^−1^ of V, Mn, Co, Ni, Cu, Zn, As, Cd and Pb above those concentrations in NASS-5. Finally, the standards as well as the samples (pH ∼1.6) were diluted 1:1 with the purified 2 M ammonium acetate buffer solution (pH 7.0) resulting in a final pH of 6.1–6.3. Polyethylene vials were used for standards and samples and were thoroughly cleaned with 10% nitric acid and Milli-Q water prior to use.

### Data analysis

Time series elution peaks were measured for each sample and standards for the trace elements evaluated (Fig. 1). The column efficiency was evaluated by calculating the trace element amount that entered the column during the pre-concentration step relatively to the total amount that was subsequently eluted. The latter was calculated based on the integrated area of the elution peak compared to the signal of a 100 μg L^−1^ solution without column. The analyte’s signals were corrected by the internal standards Ge, Rh and Ir.

In order to determine sample concentrations, an average peak count was determined over a ∼175 s period during the elution peak (a total of 25 replicate measurements). The average signal could be directly obtained from the output of Agilent’s Fileview software so no additional software was needed. The peak counts were then corrected by variations in the internal standards. The baseline levels were very low compared to the peak level and near-constant when corrected with the internals standards. Calibration curves were constructed based on the obtained response curve and no blank corrections were made. The accuracy of the quantified trace element concentrations was evaluated by measuring the CRMs SLEW-3 and CASS-4.

## Method validation

### Elution profiles, column efficiencies and recoveries

Trace elements adsorbed on the chelating resin were eluted with 5% nitric acid which were found to be adequate for elution of most the elements and has the advantage that it can be analyzed directly by ICP-MS. Elution profiles show that there were very little time-lag between the peaks for V, Mn, Ni, Co, Cu, Zn, As, Cd and Pb and baseline levels were very low compared to the peak signals (Fig. 1).

Of the trace elements studied, V, Mn, Ni, Co, Cu, Zn, Cd and Pb could be accurately measured and these elements were retained in the column and subsequently eluted resulting in column efficiencies near 100% (Table 3). With respect to As, only ∼20% of As was recovered presumably due to low affinity of the Chelex-100 resin for As, which in seawater at pH6-7 is likely to be dominant by the As(V) anion [6], [7]. Boron, Cr, Fe, Ag, Sn and Hg were either not retained or eluted adequately and could not be determined using this method. Initially, ammonium acetate buffer concentrations of 0.1, 0.2, 0.5 and 2 M were tested for sample addition but the retention and following elution of trace elements in our setup did not appear to be affected by these buffer concentrations. Consequently, the most concentrated buffer of 2 M was used in order to minimize the amount of buffer solution to be added to the acidified seawater samples to obtain a pH near 6.5. This increases the analytical signal and reduces the risk of contamination. Despite the 1:1 dilution with 2 M ammonium acetate buffer solution the pre-concentration on the column resulted in peak signal intensities in the eluent that were ∼10 times higher than if the solution had been measured directly. In contrast to direct measurement of seawater, less than ∼0.02% of Na contained in the samples were estimated to enter the instrument, salts accumulating on cones were adequately minimized, and the instrument could be used for days of analysis without cleaning.

The performance of the method was also tested for the recovery of a 10 μg L^−1^ spike to a solution containing the seawater CRM CASS-4. The recovery percentage was calculated as the concentration measured in the spiked solution relative to the theoretical value. The results show that >93% of the spike was recovered for V, Mn, Ni, Co, Cu, Zn, As, Cd and Pb (Table 3). For As, it shows that despite a column efficiency of only ∼20%, a near similar fraction of the As is retained and subsequently eluted from the spiked sample (based on CASS-4) and from the standard solutions (based on NASS-5). It indicates that As, despite a low column efficiency, can still be determined fairly accurately in different seawater matrices using this method.

### Calibration curves and detection limits

The calibration curves of V, Mn, Ni, Co, Cu, Zn, Cd, As and Pb showed good linearity with *r*^2^ > 0.999 for a NASS-5 solution spiked with 0, 0.2, 0.5, 1, 2, 5 and 10 μg L^−1^ of these elements. NASS-5 contains the lowest concentrations of most elements in any commercially available seawater CRM and therefore provides an adequate starting solution to prepare standard solutions from. Many studies have prepared standard solutions with either a Milli-Q matrix [13] or a synthetic sodium chloride matrix [4]. Standard solutions based on a Milli-Q matrix will not show signal suppression effects or other interferences caused by the seawater matrix. Standard solutions prepared with a synthetic sodium chloride matrix may suffer from traces of analytes contained in the sodium chloride added. Consequently, we chose to prepare the standard solutions from a low-trace-element-concentration seawater CRM. Only ∼2 mL sample/standard is required per measurement using the proposed method and therefore the quantity used of the rather expensive CRM is very low. Signal suppression effects and other interferences were minimized by tuning the ICP-MS to an oxide level <1% in He mode and by using a series of internal standards (Ge, Rh and Ir) with ionization potentials and masses close to those of the analytes.

For the quantitative analyses no blank correction was necessary as the standards and samples were treated in exactly the same way adding the same amount and concentration of buffer. The blank value is therefore ‘contained’ in the calibration curve. Detection limits were estimated as the concentrations corresponding to three times the standard deviation of measurements of analytes in a series of blank solutions (MilliQ water with 0.5% HNO_3_ and buffer solution) (*n* = 10) treated the same way as the samples. The results are given in Table 3. The detection limits were close to the range of previously reported values [3], [1], [14], [15] and below typical trace element concentrations observed in unpolluted open ocean seawater e.g., in NASS-5.

### Accuracy, precision and quality assurance

Trace elements in the seawater CRMs: SLEW-3 (estuarine seawater) and CASS-4 (coastal seawater) were determined using the proposed method. The analytical results for V, Mn, Ni, Co, Cu, Zn, As, Cd and Pb are listed in Table 4 together with the certified values of the CRMs. It is seen in Table 4 that experimental values for the trace elements listed agreed well with the certified values. This agreement indicates that the present method is accurate enough for the determination of trace elements in various types of seawater and that the variation between the different matrices involved has a negligible effect on the results. In this study, the period of pre-concentration and adsorption of trace elements on the column was set to 150 s but it is possible that it would be beneficial with an even longer period for very low concentration samples.

The precision of the method was evaluated by measuring a sample of CASS-4 spiked with 10 μg L^−1^ of analyte (*n* = 5). The measured precision (% RSD) was: V (0.9%), Mn (1.3%), Co (4.8%), Ni (1.7%), Cu (1.4%), Zn (0.9%), As (2.6%), Cd (1.4%) and Pb (0.7%).

For quality assurance of the method the laboratory participated in six successive rounds of the QUASIMEME intercalibration program from October 2008-August 2011. The results can be seen in Supplementary data. Out of 155 analytical results for a total of 18 seawater samples, 133 results were “Satisfactory” (within ±2 SD from the assigned mean value), two results were “Questionable” (between ±2 and ±3 SD from the assigned mean value) and none were “Unsatisfactory” (more than ±3 SD from the assigned mean value). 16 results were “Blanc”, which means that a precise concentration in the sample could not be assigned by QUASIMEME, most often due to extremely low concentrations of specific elements [12]. Four results were reported correctly as below the detection limit. The results from participation in the QUASIMEME intercalibration program document a satisfactory accuracy and long-term reliability of the proposed method.

## Additional information

Analysis of trace elements in seawater is challenging due to extremely low concentrations of most trace elements in seawater and the considerable influence of matrix elements such as Na, Mg, Ca, K and Cl. Inductively coupled plasma mass spectrometry (ICP-MS) allows direct detection of trace elements at the sub-μg L^−1^ level but spectral and non-spectral interferences caused by the seawater matrix elements limits direct determination by ICP-MS. Spectral interferences are caused by the presence of polyatomic species that interfere on the analyte masses e.g., ^35^Cl^16^O^+^ on ^51^V^+^ and ^40^Ar^23^Na^+^ on ^63^Cu^+^. Non-spectral interferences include signal suppression due to the influence of easily ionized matrix elements on the plasma (Na and K in particular) as well as signal drift caused by accumulation of salts on the cones and lenses of the ICP-MS. Simply diluting the seawater with pure water is a way of reducing the matrix effects but most often it results in inadequate instrument sensitivity, especially for open ocean seawater.

A common way of solving the analytical problem of seawater analysis is to pre-concentrate the trace elements on a chelating resin followed by a rinse of matrix elements from the resin and elution of the trace elements and detection using ICP-MS [1], [4], [14], [15]. Pre-concentration and elution can either be made ‘off-line’ with subsequent analysis of the collected eluate [3], [15], [8] or ‘on-line’ as part of the ICP-MS workflow [16], [14], [15]. Using the on-line method gives an elution profile (concentrations vs. time) of the elements eluted and usually the average peak intensity [1] or the maximum peak intensity [5] is used as proportional to the sample element concentration. The main advantage of doing the pre-concentration step on-line instead of off-line is that the contamination problems associated with the latter are greatly reduced.

The commercially available iminodiacetate chelating resin Chelex-100 has an appropriate affinity for most trace elements of interest and micro-columns filled with Chelex-100 has previously been applied for on-line pre-concentration of seawater [13]. The adsorption of trace elements on Chelex-100 is dependent on pH and has its optimum for most trace elements in seawater at around pH 6.5 [10], [11]. Since the seawater samples are usually acidified to pH 1–2 while stored, addition of a buffer, most often ammonia acetate, is used to adjust the pH of the seawater sample to pH 6–7 before being sent through the Chelex-100 filled column [3]. Previously described methods for on-line pre-concentration and analysis of seawater using ICP-MS have applied a minimum of two peristaltic pumps and/or complicated valve-systems [4], [9], [5], [14]. This requires a special (and expensive/time-consuming) instrument setup for seawater analysis differing from the standard setup used e.g. for trace element analysis in biota and sediment. The aim of this study was to find a simple and accurate way of measuring trace elements in seawater requiring as few changes to the standard ICP-MS instrument setup as possible and thereby allowing most laboratories to use the method. The performance of method was tested for trace elements included in the international QUASIMEME intercalibration program for seawater analysis [12].

## Figures and Tables

**Table 1:** Instrumental operating conditions and data acquisition settings.

	Mode of operation	
Operating conditions	Pre-concentration	Elution
RF power (W)	1550	1550
RF matching (V)	1.74	1.74
Sample depth (mm)	∼10	∼10^a^
Nebulizer, carrier gas flow (L min^−1^)	0	∼1.25^a^
Nebulizer, make up gas flow (L min^−1^)	0.30	∼0.15^a^
Collision gas (He) flow (L min^−1^)	0	∼3.5^a^
Detection mode	Spectral	Spectral
Peaks per mass	1	1
Integration time (s)	0.1	0.1
Repetitions	25	25
Masses detected	^193^Ir	^51^V, ^55^Mn, ^58^Ni, ^59^Co, ^60^Ni, ^61^Ni, ^62^Ni, ^63^Cu, ^64^Zn, ^65^Cu, ^66^Zn, ^72^Ge, ^75^As, ^103^Rh, ^111^Cd, ^113^Cd, ^193^Ir, ^206^Pb, ^207^Pb, ^208^Pb

a Adjusted daily to an oxide level <1%.

**Table 2:** Summary of the pre-concentration (steps 1 and 2) and elution (steps 3–5) program.

Step	Time (s)	Peri-pump (rps)	Flow (mL min^−1^)	Comment
1	0–150	0.2	0.8	Matrix separation and analyte pre-concentration (sample is taken up and loaded into the column)
2	150–180	0.2	0.8	Column rinsing with 0.05 M NH_4_Ac (pH 7.0)
3	180–505	0.1	0.4	Analyte elution using 5% HNO_3_ and measurement by ICP-MS
4	505–625	0.2	0.8	Column rinsing with 5% (m/m) HNO_3_
5	625–655	0.2	0.8	Column reconditioning with 0.05 M NH_4_Ac (pH 7.0) rinse solution

**Table 3:** Column efficiencies, recoveries of spikes, *r*^2^ of calibration lines and analytical detection limits.

Element (isotope)	Column efficiency (%)	Recovery of spike (%)^a^	*r*^2^ of calibration lines^b^	Detection limit^c^ (ng L^−1^)
V (51)	101 ± 4	101 ± 1	0.9992	71
Mn (55)	84 ± 3	98 ± 1	0.9998	5
Co (59)	95 ± 5	100 ± 2	0.9995	6
Ni (60)	103 ± 5	93 ± 4	0.9998	89
Cu (63)	96 ± 5	101 ± 1	0.9993	36
Zn (64)	82 ± 9	94 ± 1	0.9991	159
As (75)	19 ± 3	95 ± 3	0.9997	345
Cd (111)	81 ± 4	99 ± 1	0.9997	5
Pb (208)	81 ± 2	99 ± 1	0.9973	17

a Based on analytical signals from 5 CASS-4 samples spiked with 10 μg L^−1^ of analyte (mean ± SD).

b Determined from a series of standards where 0, 0.2, 0.5, 1, 5 and 10 μg L^−1^ of the analytes were added to a sample of the seawater CRM NASS-5.

c Calculated as the concentration corresponding to three times the standard deviation on 10 blank samples.

**Table 4:** Analytical results in μg L^−1^ for trace elements measured in seawater certified reference materials (mean ± one SD). LOD, limit of detection.

	SLEW-3		CASS-4	
	Measured (*n* = 5)	Certificate value	Measured (*n* = 5)	Certificate value
V	2.80 ± 0.06	2.57 ± 0.31	1.25 ± 0.11	1.11 ± 0.16
Mn	1.67 ± 0.04	1.61 ± 0.22	2.92 ± 0.01	2.78 ± 0.19
Ni	1.37 ± 0.06	1.23 ± 0.07	0.329 ± 0.043	0.314 ± 0.030
Co	0.033 ± 0.003	0.042 ± 0.010	0.033 ± 0.004	0.026 ± 0.003
Cu	1.63 ± 0.04	1.55 ± 0.12	0.644 ± 0.042	0.592 ± 0.055
Zn	<0.159 (LOD)	0.201 ± 0.037	0.498 ± 0.175	0.381 ± 0.057
As	1.04 ± 0.10	1.36 ± 0.09	1.28 ± 0.14	1.11 ± 0.16
Cd	0.044 ± 0.008	0.048 ± 0.004	0.020 ± 0.006	0.026 ± 0.003
Pb	<0.017 (LOD)	0.0090 ± 0.0014	<0.017 (LOD)	0.0098 ± 0.0036
