# Supplementary material for: Trace elements determination in seawater by ICP-MS with on-line pre-concentration on a Chelex-100 column using a ‘standard’ instrument setup
Source: MethodsX. 2015 Jun 18;2:323–30. doi: 10.1016/j.mex.2015.06.003 (PMC4522618; doi:10.1016/j.mex.2015.06.003)
Supplement: Supplementary file 1 [file mmc1.pdf]

Supplementary data

Trace elements determination in seawater by ICP-MS with on-line pre-concentration on a Chelex-100 micro-column using a 'standard' instrument setup.

Jens Søndergaard*, Gert Asmund, Martin M. Larsen

***corresponding author:**

Department of Bioscience, Aarhus University, Frederiksborgvej 399, DK-4000 Roskilde, Denmark

Phone: +45 87 15 87 15

E-mail: js@bios.au.dk

Table of contents:

Page 2: BIOS's parcipitation in QUASIMEME, Round 55 for seawater analysis.

Page 3: BIOS's parcipitation in QUASIMEME, Round 57 for seawater analysis.

Page 4: BIOS's parcipitation in QUASIMEME, Round 59 for seawater analysis.

Page 5: BIOS's parcipitation in QUASIMEME, Round 61 for seawater analysis.

Page 6: BIOS's parcipitation in QUASIMEME, Round 63 for seawater analysis.

Page 7: BIOS's parcipitation in QUASIMEME, Round 65 for seawater analysis.

05 February 2009

Quasimeme Database

Q104D NERI
Dr Gert Asmund
Denmark

Exercise 819 – R55 Metals in Seawater: Oct 2008 – Jan 2009

Matrix	*Determinand*	*Mean*	*Units*	*Assigned Value*	*Total Error*	*Z Score*	*z*	*Total Dupl.*
QTM133SW	Cadmium	0.017	µg/l	0.017	0.005	0.0	S	1
QTM133SW	*Cobalt*	*0.024*	*µg/l*				*B*	*1*
QTM133SW	Copper	32.71	µg/l	32.80	4.199	0.0	S	1
QTM133SW	*Iron*	*2.697*	*µg/l*				*B*	*1*
QTM133SW	Lead	0.800	µg/l	0.894	0.117	-0.8	S	1
QTM133SW	Manganese	0.225	µg/l	0.272	0.234	-0.2	S	1
QTM133SW	Nickel	0.489	µg/l	0.376	0.147	0.8	S	1
QTM133SW	Vanadium	1.380	µg/l	1.735	0.317	-1.1	S	1
QTM133SW	Zinc	9.110	µg/l	10.80	1.550	-1.1	S	1
QTM134SW	Cadmium	0.194	µg/l	0.210	0.029	-0.6	S	1
QTM134SW	*Cobalt*	*0.082*	*µg/l*				*B*	*1*
QTM134SW	Copper	34.38	µg/l	34.89	4.462	-0.1	S	1
QTM134SW	Iron	1.522	µg/l	1.462	0.383	0.2	S	1
QTM134SW	Lead	1.186	µg/l	1.330	0.171	-0.8	S	1
QTM134SW	Manganese	0.282	µg/l	0.361	0.245	-0.3	S	1
QTM134SW	Nickel	0.632	µg/l	0.596	0.175	0.2	S	1
QTM134SW	Vanadium	1.611	µg/l	2.050	0.356	-1.2	S	1
QTM134SW	Zinc	9.235	µg/l	10.90	1.562	-1.1	S	1
QTM135SW	Cadmium	0.787	µg/l	0.619	0.080	2.1	Q	1
QTM135SW	Cobalt	0.916	µg/l	0.910	0.214	0.0	S	1
QTM135SW	Copper	12.10	µg/l	12.49	1.662	-0.2	S	1
QTM135SW	Iron	1.446	µg/l	1.356	0.370	0.2	S	1
QTM135SW	Lead	0.743	µg/l	0.877	0.115	-1.2	S	1
QTM135SW	Manganese	0.828	µg/l	0.628	0.278	0.7	S	1
QTM135SW	Nickel	1.568	µg/l	1.518	0.290	0.2	S	1
QTM135SW	Vanadium	1.056	µg/l	1.314	0.264	-1.0	S	1
QTM135SW	Zinc	3.835	µg/l	4.611	0.776	-1.0	S	1

The letters in the z column indicate: S – Satisfactory, Q – Questionable,

U – Unsatisfactory, C – Consistent, I – Inconsistent, B - Blanc.

If the analytical value looks like this, then the Assigned value is indicative only.

01 October 2009

Quasimeme Database

Q104D NERI
Dr Gert Asmund
Denmark

Exercise 837 – R57 Metals in Seawater: Apr – Jul 2009

Matrix	*Determinand*	*Mean*	*Units*	*Assigned Value*	*Total Error*	*Z Score*	*z*	*Total Dupl.*
QTM139SW	Arsenic	2.086	µg/l	1.235	0.404	2.1	Q	1
QTM139SW	Cadmium	<0.031	µg/l	0.015	0.004		C	1
QTM139SW	*Cobalt*	*0.206*	*µg/l*				*B*	*1*
QTM139SW	Copper	1.574	µg/l	2.013	0.352	-1.2	S	1
QTM139SW	Lead	0.159	µg/l	0.191	0.029	-1.1	S	1
QTM139SW	*Manganese*	*<0.033*	*µg/l*				*B*	*1*
QTM139SW	Nickel	0.980	µg/l	0.961	0.220	0.1	S	1
QTM139SW	*Silver*	*<0.012*	*µg/l*				*B*	*1*
QTM139SW	*Tin*	*<1.050*	*µg/l*				*B*	*1*
QTM139SW	Vanadium	1.034	µg/l	1.222	0.253	-0.7	S	1
QTM139SW	Zinc	1.476	µg/l	1.430	0.379	0.1	S	1
QTM140SW	Arsenic	3.441	µg/l	2.829	0.604	1.0	S	1
QTM140SW	Cadmium	0.306	µg/l	0.377	0.050	-1.4	S	1
QTM140SW	Cobalt	0.349	µg/l	0.161	0.120	1.6	S	1
QTM140SW	Copper	2.245	µg/l	2.916	0.465	-1.4	S	1
QTM140SW	Lead	0.595	µg/l	0.635	0.084	-0.5	S	1
QTM140SW	Manganese	0.968	µg/l	1.477	0.385	-1.3	S	1
QTM140SW	Nickel	1.743	µg/l	1.774	0.322	-0.1	S	1
QTM140SW	Silver	0.147	µg/l	0.224	0.128	-0.6	S	1
QTM140SW	*Tin*	*<1.050*	*µg/l*				*B*	*1*
QTM140SW	Vanadium	2.078	µg/l	2.444	0.406	-0.9	S	1
QTM140SW	Zinc	7.513	µg/l	7.835	1.179	-0.3	S	1
QTM141SW	Arsenic	3.232	µg/l	2.800	0.600	0.7	S	1
QTM141SW	Cadmium	0.914	µg/l	0.883	0.113	0.3	S	1
QTM141SW	Cobalt	1.478	µg/l	1.353	0.269	0.5	S	1
QTM141SW	Copper	1.842	µg/l	2.157	0.370	-0.9	S	1
QTM141SW	Lead	0.363	µg/l	0.398	0.055	-0.6	S	1
QTM141SW	Manganese	0.762	µg/l	0.739	0.292	0.1	S	1
QTM141SW	Nickel	0.721	µg/l	0.682	0.185	0.2	S	1
QTM141SW	Silver	0.914	µg/l	0.687	0.186	1.2	S	1
QTM141SW	*Tin*	*<1.050*	*µg/l*				*B*	*1*
QTM141SW	Vanadium	0.971	µg/l	1.053	0.232	-0.4	S	1
QTM141SW	Zinc	3.483	µg/l	3.692	0.662	-0.3	S	1

11 February 2010

Quasimeme Database

Q104D NERI
Dr Gert Asmund
Denmark

Exercise 864 – R59 Metals in Seawater: Oct 2009 – Jan 2010

Matrix	*Determinand*	*Mean*	*Units*	*Assigned Value*	*Total Error*	*Z Score*	*z*	*Total Dupl.*
QTM145SW	Cadmium	<0.080	µg/l	0.013	0.004		I	1
QTM145SW	*Cobalt*	*<0.010*	*µg/l*				*B*	*1*
QTM145SW	Copper	9.131	µg/l	9.401	1.275	-0.2	S	1
QTM145SW	Lead	0.257	µg/l	0.266	0.083	-0.1	S	1
QTM145SW	Manganese	0.229	µg/l	0.337	0.242	-0.4	S	1
QTM145SW	Nickel	0.282	µg/l	0.322	0.140	-0.3	S	1
QTM145SW	*Silver*	*<0.358*	*µg/l*				*B*	*1*
QTM145SW	Vanadium	1.546	µg/l	1.668	0.308	-0.4	S	1
QTM145SW	Zinc	12.94	µg/l	12.47	1.759	0.3	S	1
QTM146SW	Cadmium	0.230	µg/l	0.297	0.040	-1.7	S	1
QTM146SW	Cobalt	0.134	µg/l	0.132	0.116	0.0	S	1
QTM146SW	Copper	5.266	µg/l	5.695	0.812	-0.5	S	1
QTM146SW	Lead	0.551	µg/l	0.576	0.122	-0.2	S	1
QTM146SW	Manganese	0.883	µg/l	1.062	0.333	-0.5	S	1
QTM146SW	Nickel	1.211	µg/l	1.410	0.276	-0.7	S	1
QTM146SW	*Silver*	*<0.358*	*µg/l*				*B*	*1*
QTM146SW	Vanadium	2.344	µg/l	2.431	0.404	-0.2	S	1
QTM146SW	Zinc	11.41	µg/l	10.75	1.543	0.4	S	1
QTM147SW	Cadmium	0.883	µg/l	0.852	0.109	0.3	S	1
QTM147SW	Cobalt	1.230	µg/l	1.277	0.260	-0.2	S	1
QTM147SW	Copper	3.492	µg/l	3.918	0.590	-0.7	S	1
QTM147SW	Lead	0.497	µg/l	0.563	0.120	-0.6	S	1
QTM147SW	Manganese	0.827	µg/l	0.814	0.302	0.0	S	1
QTM147SW	Nickel	0.508	µg/l	0.623	0.178	-0.6	S	1
QTM147SW	Silver	0.545	µg/l	0.508	0.163	0.2	S	1
QTM147SW	Vanadium	1.046	µg/l	1.035	0.229	0.0	S	1
QTM147SW	Zinc	4.842	µg/l	4.927	0.816	-0.1	S	1

The letters in the z column indicate: S – Satisfactory, Q – Questionable,

U – Unsatisfactory, C – Consistent, I – Inconsistent, B - Blanc.

If the analytical value looks like this, then the Assigned value is indicative only.

22 September 2010

Quasimeme Database

Q104D NERI
Dr Gert Asmund
Denmark

Exercise 882 – R61 Metals in Seawater: Apr – Sep 2010

Matrix	*Determinand*	*Mean*	*Units*	*Assigned Value*	*Total Error*	*Z Score*	*z*	*Total Dupl.*
QTM151SW	Cadmium	0.018	µg/l	0.016	0.004	0.4	S	1
QTM151SW	*Cobalt*	*0.011*	*µg/l*				*B*	*1*
QTM151SW	Copper	2.880	µg/l	2.794	0.449	0.2	S	1
QTM151SW	Lead	0.147	µg/l	0.134	0.022	0.6	S	1
QTM151SW	Manganese	0.160	µg/l	0.236	0.230	-0.3	S	1
QTM151SW	Nickel	0.319	µg/l	0.351	0.144	-0.2	S	1
QTM151SW	Vanadium	1.640	µg/l	1.622	0.303	0.1	S	1
QTM151SW	Zinc	8.960	µg/l	9.590	1.399	-0.5	S	1
QTM152SW	Cadmium	0.600	µg/l	0.559	0.072	0.6	S	1
QTM152SW	Cobalt	0.255	µg/l	0.239	0.130	0.1	S	1
QTM152SW	Copper	4.710	µg/l	4.348	0.644	0.6	S	1
QTM152SW	Lead	1.170	µg/l	1.022	0.133	1.1	S	1
QTM152SW	Manganese	2.300	µg/l	2.181	0.473	0.3	S	1
QTM152SW	Nickel	2.160	µg/l	2.230	0.379	-0.2	S	1
QTM152SW	Vanadium	3.150	µg/l	2.927	0.466	0.5	S	1
QTM152SW	Zinc	11.70	µg/l	11.77	1.672	0.0	S	1
QTM153SW	Cadmium	1.240	µg/l	1.133	0.144	0.7	S	1
QTM153SW	Cobalt	1.600	µg/l	1.549	0.294	0.2	S	1
QTM153SW	Copper	4.170	µg/l	3.804	0.576	0.6	S	1
QTM153SW	Lead	0.957	µg/l	0.829	0.109	1.2	S	1
QTM153SW	Manganese	1.640	µg/l	1.579	0.397	0.2	S	1
QTM153SW	Nickel	1.280	µg/l	1.319	0.265	-0.1	S	1
QTM153SW	Vanadium	1.950	µg/l	1.882	0.335	0.2	S	1
QTM153SW	Zinc	7.110	µg/l	6.960	1.070	0.1	S	1

The letters in the *z* column indicate: S – Satisfactory, Q – Questionable, U – Unsatisfactory, C – Consistent, I – Inconsistent, B - Blanc.

If the analytical value looks like this, then the Assigned value is indicative only.

QUASIMEME Project Office
Wageningen UR
P.O. Box 47
6700 AA Wageningen
The Netherlands
Tel.: +31 (0) 317 486 546
Fax: +31 (0) 317 426 101
Email: quasimeme@wur.nl
www.quasimeme.org

14 February 2011

Quasimeme Database

Q104D NERI
Dr Gert Asmund
Denmark

Exercise 906 – R63 Metals in Seawater: Oct 2010 – Jan 2011

Matrix	*Determinand*	*Mean*	*Units*	*Assigned Value*	*Total Error*	*Z Score*	*z*	*Total Dupl.*
QTM157SW	Arsenic	1.332	µg/l	1.274	0.409	0.1	S	1
QTM157SW	Cadmium	<0.013	µg/l	0.011	0.004		C	1
QTM157SW	*Cobalt*	*0.011*	*µg/l*				*B*	*1*
QTM157SW	Copper	3.630	µg/l	3.671	0.559	-0.1	S	1
QTM157SW	Manganese	0.274	µg/l	0.305	0.238	-0.1	S	1
QTM157SW	Vanadium	1.462	µg/l	1.564	0.295	-0.3	S	1
QTM158SW	Arsenic	4.498	µg/l	3.716	0.715	1.1	S	1
QTM158SW	Cadmium	0.694	µg/l	0.722	0.093	-0.3	S	1
QTM158SW	Cobalt	0.389	µg/l	0.403	0.150	-0.1	S	1
QTM158SW	Copper	4.898	µg/l	5.027	0.728	-0.2	S	1
QTM158SW	Lead	0.936	µg/l	0.946	0.123	-0.1	S	1
QTM158SW	Manganese	2.192	µg/l	2.403	0.500	-0.4	S	1
QTM158SW	Vanadium	2.746	µg/l	2.892	0.462	-0.3	S	1
QTM159SW	Arsenic	4.969	µg/l	3.997	0.750	1.3	S	1
QTM159SW	Cadmium	1.064	µg/l	1.031	0.131	0.3	S	1
QTM159SW	Cobalt	0.555	µg/l	0.553	0.169	0.0	S	1
QTM159SW	Copper	3.513	µg/l	3.209	0.501	0.6	S	1
QTM159SW	Lead	1.205	µg/l	1.226	0.158	-0.1	S	1
QTM159SW	Manganese	2.897	µg/l	3.049	0.581	-0.3	S	1
QTM159SW	Vanadium	2.353	µg/l	2.497	0.412	-0.3	S	1

The letters in the z column indicate: S – Satisfactory, Q – Questionable,

U – Unsatisfactory, C – Consistent, I – Inconsistent, B - Blanc.

If the analytical value looks like this, then the Assigned value is indicative only.

QUASIMEME Project Office
Wageningen UR
P.O. Box 47
6700 AA Wageningen
The Netherlands
Tel.: +31 (0) 317 486 546
Fax: +31 (0) 317 426 101
Email: quasimeme@wur.nl
www.quasimeme.org

05 September 2011

Quasimeme Database

Q104D NERI
Dr Gert Asmund
Denmark

Exercise 924 – R65 Metals in Seawater: Apr – Aug 2011

Matrix	*Determinand*	*Mean*	*Units*	*Assigned Value*	*Total Error*	*Z Score*	*z*	*Total Dupl.*
QTM163SW	Cadmium	<0.019	µg/l	0.015	0.004		C	1
QTM163SW	*Cobalt*	*0.039*	*µg/l*				*B*	*1*
QTM163SW	Copper	17.70	µg/l	17.36	2.270	0.1	S	1
QTM163SW	Lead	0.360	µg/l	0.316	0.045	1.0	S	1
QTM163SW	Manganese	0.189	µg/l	0.274	0.234	-0.4	S	1
QTM163SW	Nickel	0.243	µg/l	0.289	0.136	-0.3	S	1
QTM163SW	Vanadium	1.520	µg/l	1.591	0.299	-0.2	S	1
QTM163SW	Zinc	12.10	µg/l	11.64	1.654	0.3	S	1
QTM164SW	Cadmium	0.021	µg/l	0.025	0.006	-0.7	S	1
QTM164SW	*Cobalt*	*0.077*	*µg/l*				*B*	*1*
QTM164SW	Copper	18.00	µg/l	17.44	2.280	0.2	S	1
QTM164SW	Lead	0.426	µg/l	0.402	0.055	0.4	S	1
QTM164SW	Manganese	0.331	µg/l	0.388	0.249	-0.2	S	1
QTM164SW	Nickel	0.528	µg/l	0.587	0.173	-0.3	S	1
QTM164SW	Vanadium	1.650	µg/l	1.712	0.314	-0.2	S	1
QTM164SW	Zinc	13.20	µg/l	12.63	1.779	0.3	S	1
QTM165SW	Cadmium	0.210	µg/l	0.189	0.026	0.8	S	1
QTM165SW	Cobalt	0.462	µg/l	0.437	0.155	0.2	S	1
QTM165SW	Copper	8.970	µg/l	8.106	1.113	0.8	S	1
QTM165SW	Lead	1.150	µg/l	1.059	0.137	0.7	S	1
QTM165SW	Manganese	2.260	µg/l	2.231	0.479	0.1	S	1
QTM165SW	Nickel	3.850	µg/l	3.625	0.553	0.4	S	1
QTM165SW	Vanadium	2.520	µg/l	2.485	0.411	0.1	S	1
QTM165SW	Zinc	12.10	µg/l	10.30	1.487	1.2	S	1

The letters in the z column indicate: S – Satisfactory, Q – Questionable,

U – Unsatisfactory, C – Consistent, I – Inconsistent, B - Blanc.

If the analytical value looks like this, then the Assigned value is indicative only.
